# The Influence of Wool Pellet Application on Alleviating Salt-Induced Stress in Soybean (*Glycine max* L.)

**DOI:** 10.3390/life15030328

**Published:** 2025-02-20

**Authors:** Lütfi Nohutçu

**Affiliations:** Department of Field Crops, Faculty of Agriculture, Van Yüzüncü Yıl University, 65090 Van, Türkiye; lutfinohutcu@yyu.edu.tr

**Keywords:** growth indices, malondialdehyde, MDA, salt stress, wool pellets

## Abstract

Soil salinity is a pervasive challenge in agricultural regions, significantly impacting plant growth and productivity. Addressing the adverse implications of soil salinization and capitalizing on organic waste resources has the potential to yield substantial advancements in the agricultural sector. This study examined the influence of varying levels of wool pellets (0, 1%, 2%, and 4%) and salt (0, 25, 50, and 100 mM) on the physiological and biochemical properties of soybean (*Glycine max* L.). The findings revealed that compared to the control group, at a 4% application rate, plant length decreased by 20%, while stem dry weight, root length and weight showed no significant differences up to a 2% application. Compared to the control treatment, the 4% application rate resulted in an increase of 6% in leaf temperature, 55% in NBI, 12% in chlorophyll content, and 10% in MDA activity. Conversely, the TAA, TPC, and carotenoid content decreased by 55%, 51%, and 81%, respectively. Salt applications led to reductions in most studied morphological parameters, except for root properties. Compared to the control, plant length, stem fresh weight, and stem dry weight decreased by 14%, 22%, and 14%, respectively, while root length, root fresh weight, and root dry weight increased by 18%, 33%, and 50%, respectively.

## 1. Introduction

Every year, an enormous amount of organic waste and byproducts are produced all over the world. Slaughterhouses, meat and poultry farms, and wool textile industries generate large amounts of fibrous protein-rich wastes, such as collagen, elastin, and keratin, which require proper management [1,2]. Wool is an eco-friendly fiber [3]. Wool fiber exhibits a unique and complex structure, with a varied chemical composition that distinguishes it from other plant-based fibers. Its diverse characteristics, heterogeneous properties, and advantageous qualities set it apart from both natural and synthetic fibers [4]. Wool was the most valued sheep product for a long period and generated the highest income for the farmers [5]. Wool prices decreased significantly, and wool manufacturers lost profitability throughout the past two decades due to the textile industry’s preference for artificial textile materials and fibers. Wool contains 1% non-protein material, 17% non-keratin protein, and 82% keratin protein [6].

The salinization of soil is attributable to the accumulation of soluble salts, such as sodium chlorides and sulfates, with more than 3% of agricultural lands being significantly impacted by this phenomenon [7,8]. Increased sodium ion concentrations in the soil elevate osmotic pressure, disrupt cellular ion balance, and impede water and nutrient absorption, consequently hindering soybean growth and diminishing agricultural yields [9]. Salt stress prompts the generation of reactive oxygen species, which serve as secondary stress agents and instigate membrane lipid peroxidation, culminating in the degradation of the structural integrity of cell membrane proteins [10]. Moreover, high sodium ion (Na^+^) concentrations impair the uptake of potassium ions (K^+^), an essential element for plant growth and development [11]. A reduction in potassium absorption led to a significant decrease in the potassium-to-sodium ratio within the plant tissue, corresponding to the NaCl concentration in the nutrient solution [12]. Fertilization is crucial for achieving high efficiency in plant production and maintaining productivity. While chemical fertilizers and pesticides can control plant pests and provide insect protection, their prolonged use can harm soil microorganisms. Soybean was chosen as the model plant in this study due to its widespread cultivation and diverse applications across various sectors as a raw material. The basis for choosing sheep wool manure was its evaluation as an organic, slow-release source that serves as an essential nutrient for various crops. The research was designed with the hypothesis that this waste product could supply crops with the necessary nutrients for sustainable agriculture [13]. Sheep wool waste has been identified as a valuable nutrient source for agricultural fertilizers. Research has shown that wool protein hydrolyzates can provide a good supply of nitrogen for plant growth beyond just the disposal of wool waste materials [14]. Wool blocks and granules are completely natural and biodegradable materials that are regularly used in soil for nutrient fertilization and have no negative environmental impact. They cause no soil pollution, water contamination, or soil degradation while protecting biodiversity and preventing hazardous compounds from infiltrating groundwater or soil surface [15].

This study aimed to investigate the effect of wool pellets obtained from waste sheep wool on reducing salt stress in soybean’s (*Glycine max* L.) physiological and biochemical properties.

## 2. Materials and Methods

### 2.1. Material

This study was carried out in January 2024 in a growth chamber at the Department of Field Crops, Faculty of Agriculture, Van Yuzuncu Yil University (Van YYU). Soybean seeds (*Glycine max* L.-ANP-2018 variety) were planted in 500 cc pots in a mixture of peat + perlite + soil (1:1:2) and different rates of wool pellets as 0%, 1%, 2%, and 4%, then kept in a growth chamber (65% RH; 8/16 h dark/light intervals; 25 °C). Wool pellets were obtained from Woolpell^©^ company (Kahramankazan, Turkey) and were made of 100% organic material. Table 1 shows the content of wool pellets. When they had 5–6 leaves (42nd day), the cultivated plants were subjected to salt stress (NaCl) by irrigation at the rate of 0, 25, 50, and 100 mM. The plants were watered with 50 mL of water every two days. Physiological problems were observed in plants approximately on the 50th day after three applications of salt. This study was terminated by harvesting for analyses.

### 2.2. Methods

The plant length was measured from the soil surface to the top of the plant using digital calipers, and the length of the roots was measured with digital calipers from the soil surface to the tip of the root after the plants were carefully extracted and cleaned. After measurement, the fresh and dry weights of the root and stem of the plant were determined with the help of a precision scale (0.0001 g). The plants and roots, whose fresh weights were determined, were then dried at 40 °C for 72 h and their dry weights were determined. The leaf area index was determined using the Easy Leaf Area software (version 2.0) (6 March 2024), and the leaf surface temperatures were measured with a portable infrared thermometer. Nitrogen balance index (NBI), chlorophyll, and flavonoid content in leaves were measured non-destructively and in real time on the leaf with the Dualex scientific+ (FORCE-A, Orsay, France) device [16].

The level of lipid peroxidation was measured based on a malondialdehyde (MDA) content assay [17]. For this purpose, leaf samples (0.5 g) were homogenized in 10 mL of 0.1% trichloroacetic acid (TCA), followed by centrifugation at 15,000 rpm for 5 min. Then, 1.0 mL of the supernatant was mixed with 4.0 mL of 0.5% thiobarbituric acid (TBA) in 20% TCA. The mixture was heated at 95 °C for 30 min and then quickly cooled in ice. After centrifugation at 10,000 rpm for 10 min, the absorbance of the supernatant was recorded at 532 nm and 600 nm. Total phenolic compound content was measured using the Obanda, Owuor method [18]. The antioxidant activity was also performed based on the antioxidant power (FRAP) (Iron (III) antioxidant power reduction) method [19], followed by readings of the absorbance at 593 nm and antioxidant activity values were recorded as Trolox equivalent (TE)/mg. Carotenoid pigments were extracted from the leaves using 100% acetone and a mortar and pestle. Absorbance of the extracts was measured at wavelengths of 470 nm, 645 nm, and 662 nm using a UNICAM 8625 spectrophotometer. Total carotenoid concentrations were then calculated employing the equations developed by Lichtenthaler and Wellburn [20].

The data obtained in this study were subjected to variance analysis according to the factorial order in the randomized plots experimental design with the help of the Costat (version 6.34) package program, and the averages were calculated using the LSD multiple comparison test [21].

## 3. Results

The results showed significant effects of wool pellet treatments on several physiological and biochemical growth parameters of soybean (*G. max* L.) under salt stress (Table 2 and Table 3). The research data indicated that the effects of wool pellet and salt treatments on soybean plant length were statistically significant, while the interaction between these two factors was not significant (*p* < 0.05, *p* < 0.01). As a result of this study, the highest plant length mean was obtained from both the control group of wool pellets (29.18 cm) and salt (28.37 cm). For both treatments, the plant length mean decreased gradually with increasing doses.

This study found that the effects of wool pellet and salt applications on root length were significant at the 1% and 5% levels, respectively. The highest root lengths were observed in the control, 1%, and 2% wool pellet treatments, while the lowest value of 21.43 cm was recorded in the 4% wool pellet treatment. Salt stress also had a significant impact on root length, with the highest value of 27.87 cm observed at a salt concentration of 100 mM and the lowest of 23.43 cm in the control.

While the effect of salt and WP × S applications on the fresh stem weight of the plant was found to be statistically significant, the effect of wool pellets was found to be insignificant (*p* < 0.01). As a result of four different salt applications in the research, the lowest stem fresh weight was measured in the 100 mM salt application with 1.15 g, while the other salt applications (0, 25, 50 mM) were in the same group.

The impact of wool pellets, salt, and WP × S treatments on the plant’s root fresh weight was found to be statistically significant. This study examined four different wool pellet application rates, and the results showed that the highest stem fresh weight was observed in the control, 1%, and 2% treatments, while the lowest value was recorded in the 4% treatment. Also, it can be seen in Table 2 that with a salt application, the highest root fresh weight was observed at 100 mM with 1.43 g, while the lowest results were obtained in the control and 25 mM salt applications with 1.07 g and 1.15 g, respectively. It is observed that the root fresh weight increased with the increase in salt doses.

The results presented in Table 2 indicate that the combination of wool pellets, salt doses, and their interaction had a statistically significant impact on stem dry weight. Specifically, the highest stem dry weight was observed in the control group and the 1% and 2% wool pellet treatments, while the lowest value was recorded in the 4% wool pellet treatment at 0.31 g. For salt doses, the highest stem dry weight was observed in the control group at 0.50 g, with lower values observed in the other treatments. Similar to the findings for stem fresh weight, stem dry weight decreased as salt doses increased.

The effect of WP, S, and WP × S applications on the root dry weight of the plant was found to be statistically significant (*p* < 0.01). The highest root dry weight was obtained in the wool pellet control and the 1% and 2% dose applications, and the lowest value was obtained at 4%. Similar to the fresh root weight parameter, root dry weight decreased with increasing doses of salt. The highest value was obtained in 100 mM doses of salt with 0.21 g, and the lowest value was in the control with 0.14 g.

The results of this study demonstrated that salt and WP × S treatments had no statistically significant impact on leaf temperature, whereas the application of wool pellets exhibited a significant effect. The highest leaf temperature was observed in the 2% and 4% wool pellet applications, with 21.71 °C and 21.37 °C, respectively. The lowest temperature was obtained in the control with 20.08 °C (Table 3).

The effect of all treatments on leaf area was significant at the 1% level. The highest leaf area obtained as a result of wool pellet applications was 10.23 cm^2^ in the 1% dose, and the lowest was 7.98 cm^2^ in the control. Salt doses caused stress on the plant, and the leaf area was significantly affected by salt stress (*p* < 0.01). As a result of salt treatments, the highest leaf area was observed in the control with 11.79 cm^2^, while the leaf area gradually decreased with increasing doses, and the lowest area was observed at the 100 mM salt dose with 6.79 cm^2^.

The effects of wool pellets, salt, and WP × S applications on the nitrogen balance index (NBI) were found to be statistically significant (*p* < 0.01). While the lowest NBI value was obtained from the control group of wool pellet application with 81.66 dx, the highest value was determined as 126.50 dx with the 4% application. As a result of salt treatments, the highest NBI was observed in the control with 114.39 dx, while the NBI gradually decreased with increasing stress levels, and the lowest NBI was observed at the 100 mM salt dose with 82.97 dx.

The effects of applications on chlorophyll content were statistically significant (Table 3). In this study, the highest chlorophyll content was determined at 1%, 2%, and 4% of wool pellet applications. As a result of salt treatments, the highest value obtained was 42.61 dx in the control and other applications in the same group.

This study found that wool pellet and salt treatments significantly influenced the flavonoid levels in the soybean plant, whereas the combined application of these treatments did not have a significant impact (Table 4). The highest flavonoid content of wool pellet treatment was observed in all applications except for the 4% application, while the highest flavonoid content in the salt treatment was observed at the 100 mM application with 0.45 dx. It is observed that flavonoid content increases with increasing stress doses. It is known that they become active under biotic and abiotic stress conditions and protect plants.

This study found that wool pellet and salt treatments significantly influenced the total antioxidant activity (TAA) in the soybean plant, whereas the combined application of these treatments did not have a significant impact. The highest TAA of wool pellet treatment was observed in the control applications, while the highest TAA content was observed in the salt treatment at the 100 mM application with 13.31 µmol TE/g. The results indicate that total antioxidant activity exhibits an increasing trend with elevated stress levels, and the application of wool pellets appears to mitigate this stress response.

The effect of all treatments on total phenolic content (TPC) was significant at the 1% level. The highest TPC obtained as a result of wool pellet applications was 129.16 mg GAE/g in the control dose, and the lowest was 62.91 and 63.80 mg GAE/g in the 2% and 4%, respectively. As a result of salt treatments, the lowest TPC was observed in the control with 83.62 mg GAE/g, while the TPC gradually increased with increasing doses, and the highest value was observed at 100 mM salt dose with 96.53 mg GAE/g.

The results of this study demonstrated that wool pellets, salt, and their combined application had a statistically significant impact on carotenoid concentrations. The highest carotenoid levels were observed in the control group with wool pellet application, reaching 2.29 μg/g FW. In contrast, the lowest carotenoid levels were recorded at 2% and 4% salt concentrations, both registering 0.43 μg/g FW. The results of this study demonstrated that increased salt concentrations led to elevated carotenoid levels, with the lowest value observed in the control treatment (0.43 μg/g FW) and the highest carotenoid concentrations obtained under the 100 mM salt application (1.51 μg/g FW).

The effect of wool pellets, salt, and WP × S treatment on lipid peroxidation (malondialdehyde (MDA)) was found to be statistically significant at the 1% level (Table 3). The highest MDA value was obtained at different rates of wool pellet treatment, and the lowest value was obtained in the control. For the salt treatments, the highest MDA value was obtained on 100 mM salt treatment with 25.19 nmol/g FW, while the lowest value was obtained in the control with 18.88 nmol/g FW. The lipid peroxidation (MDA) content of the plant was in parallel with stress conditions, so it can also be said that it acts as the plant’s defense mechanism.

## 4. Discussion

Nitrogen is essential for plants to thrive and fulfill their life cycle [22,23,24]. It is the primary nutrient that constrains plant growth and development after carbon, hydrogen, and oxygen have participated in photosynthesis, hormone regulation, and protein changes. Sheep wool is an environmentally friendly fiber [3], and it contains 44% carbon and 10–11% nitrogen, which makes it an important C and N resource that may be used as both a soil amendment and a source of nutrients. Waste wool is also an organic waste that is not beneficial for sheep farmers because of a lack of demand. As a result, it is no longer a commercially viable product, and its safe disposal is crucial for farmers and consumers [25]. Its use as manure in soil can be a viable option [26]. Wool pellets and granules are completely natural and biodegradable materials that are routinely used in soil for nitrogen fertilization and have no negative environmental impact. They cause no soil pollution, water contamination, or soil degradation while protecting biodiversity and preventing hazardous compounds from infiltrating groundwater or soil surface [15]. Many studies have revealed that wool manure or fertilizer made from waste wool has many benefits for plant growth and yield [27,28,29,30], organic farming [31,32,33] and improving soil properties [34,35]. Previous research has shown that the application of wool pellets resulted in substantial increases in the yield and quality of both spinach and tomato crops compared to control and standard fertilization practices. Specifically, spinach yields were observed to increase by 148% and 53%, while tomato yields increased by 86% and 31%, respectively [32]. The published literature has also reported that the use of waste wool can result in significant improvements in crop productivity. These studies have documented yield increases of up to 50% for barley [28], 12% for pepper [35], and up to 15% for asparagus [36].

The present study showed that physiological and biochemical properties, such as stem and root length and weight, leaf temperature and area, nitrogen balance index, chlorophyll, flavonoid, and MDA content of soybean affect different rates of wool pellets and salt doses (Table 2 and Table 3). Plant length, stem fresh, and dry weight decrease with increasing wool pellets and salt doses. This decrease might be due to the role of salt; the results of this study and previous studies [37,38] show that salt stress has a detrimental effect on soybeans. In addition, root length and fresh and dry weight decrease with increasing wool pellets, while they increase with increasing salt doses. This might be due to a lack of soluble water around the rhizosphere, causing that plant to promote root growth to access water and nutrients. Growth and development generally negatively affect plants under salt stress, but in some cases, a plant’s reaction to salt can be reversed, as shown in previous studies [39].

Leaf temperature is influenced by various parameters, including environmental, genetic, and stress-related factors. The surface temperature of the leaf typically increases linearly with rising stress levels. Lower leaf temperatures can be advantageous in certain scenarios, such as during water scarcity, as it helps the plant maintain its water and thermal balance, thereby promoting more efficient growth and enhancing resistance to environmental stresses [40]. Elevated leaf temperatures can be advantageous in certain contexts, such as in cooler environments or for promoting accelerated seedling development [41]. Conversely, research has shown that salt stress does not significantly impact leaf temperature, whereas increasing the application of wool pellets leads to a notable rise in leaf temperature. Leaf area is an important parameter of a plant’s photosynthetic capacity, and the lack of fluctuation in this parameter could indicate that factors other than water availability play a key part in leaf development [41]. A reduction in leaf area is a typical response to stress, so the results of this research show that increasing salt doses caused a loss of leaf area. Nitrogen balance index (NBI) and chlorophyll content are increased by wool pellet doses and decreased by increasing salt doses. The nitrogen balance index parameters were utilized to assess the changes in the amount of nitrogen absorbed by the plants from their surrounding environment [42]. Consistent with our findings, previous research has shown that the soybean nitrogen balance index and chlorophyll content decrease with increasing soil salinity levels [43]. Flavonoid content and MDA (malondialdehyde) activities increased with increasing salt doses. This increase may be due to the inhibition of water uptake due to salt stress and the occurrence of drought stress in the plant. Sarker and Oba’s research [44,45,46] found that increasing levels of salt stress led to corresponding rises in the polyphenol and flavonoid content, as well as the overall antioxidant activity of the plant samples. The researchers attributed these enhancements to the salt-induced increases in the concentrations of phenolic acids and flavonoid compounds, which in turn drove the observed elevation in total antioxidant capacity. Increased accumulation of phenolic and flavonoid compounds in plant cells under salinity stress may bolster the plant’s capacity to mitigate the detrimental effects of salinity-induced oxidative stress [47,48]. Consistent with the findings of this study, previous research has reported that increased salt stress leads to a rise in flavonoid content [49] and malondialdehyde activities [50].

Salt stress stimulates the generation of a diverse array of free radicals throughout the plant, disrupting numerous biochemical and metabolic processes. This can ultimately lead to reduced yield or even the death of the entire plant in severe cases [51]. The plant’s antioxidant capacity serves as a protective mechanism against the damaging effects of free radicals. Increased stress levels lead to the upregulation of various biochemical attributes, such as antioxidants, total phenolic content, and malondialdehyde, as a defense mechanism against the adverse effects of stress. Our findings indicate that total antioxidant activity increased with escalating salt stress levels, whereas it decreased with rising wool pellet applications. Previous research on soybean under various salt stress levels has shown that increased stress leads to elevated antioxidant enzyme activities, including superoxide dismutase (SOD), catalase (CAT), ascorbate peroxidase (APX), and glutathione reductase (GR) [52,53,54].

Numerous studies have found a strong positive correlation between total phenolic content and antioxidant activity, suggesting that phenolics are major contributors to the antioxidant capacity of plants [55,56]. As expected, in our study, total antioxidant activity and total phenolic content showed similar trends. The total phenolic content was observed to decline as the application of wool pellets increased, whereas it exhibited an upward trend in response to escalating salt concentrations. According to a previous study examining seven soybean genotypes, the phenolic content of the plants increased as the stress levels were elevated [56]. Another investigation involving three soybean varieties and four contrasting salt application rates demonstrated that total phenolic content escalated concomitantly with heightened levels of salt stress [57]. The results demonstrate a positive relationship between the antioxidant activity and phenolic content of the plant. Both parameters have been observed to increase in response to elevated stress levels, as corroborated by the current findings as well as previous studies.

The chlorophyll and total carotenoid levels in leaves generally decrease under saline conditions [58]. However, some studies conducted with different plant species have reported contradictory findings regarding this phenomenon [59,60,61]. The findings reported in this study and the results obtained here do not fully align with the general information presented. There appear to be notable deviations from the established understanding of this subject matter. This study found that the carotenoid content in soybeans increased in parallel with the level of stress, exhibiting up to a threefold enhancement.

## 5. Conclusions

The findings of this study suggest that wool pellet treatments have a positive impact on various soybean parameters while also exhibiting both beneficial and detrimental effects on certain properties. Compared to the control group, at a 4% application rate, plant length decreased by 20%, while stem dry weight, root length, and root fresh and dry weight showed no significant differences up to a 2% application. Compared to the control treatment, the 4% application rate resulted in an increase of 6% in leaf temperature, 55% in NBI, 12% in chlorophyll content, and 10% in MDA activity. Conversely, the TAA, TPC, and carotenoid content decreased by 55%, 51%, and 81%, respectively. Salt applications led to reductions in most studied morphological parameters, except for root properties. Compared to the control, plant length, stem fresh weight, and stem dry weight decreased by 14%, 22%, and 14%, respectively, while root length, root fresh weight, and root dry weight increased by 18%, 33%, and 50%, respectively. Salt doses had no effect on leaf temperature, but leaf area, NBI, and chlorophyll content decreased gradually, with reductions of 42%, 27%, and 16%, respectively, compared to the control. The total antioxidant capacity, malondialdehyde activity, total phenolic content, total flavonoid content, and total carotenoid content all demonstrated marked increases in response to elevated salt concentrations. Specifically, these parameters increased by 32%, 33%, 15%, 28%, and 251%, respectively, with increasing salt exposure. The results suggest that wool pellets have a positive effect on many parameters and may have a healing and regulating effect against salt stress. However, to obtain more realistic results, further field-based testing is recommended.

## Figures and Tables

**Table 1:** Content of wool pellets (Woolpell^©^).

Organic Matter	70–83 (%)
Nitrogen (N)	7–9 (%)
Phosphorus (P)	0.4 (%)
pH	5 (%)
Humic + Fulvic Acid	9–11
Calcium (Ca)	42 (%)
Magnesium (Mg)	0.4 (%)
Iron (Fe)	400 (mg kg^−1^)
Zinc (Zn)	90 (mg kg^−1^)

**Table 2:** The effect of wool pellets and salt doses on morphological parameters of soybean.

Wool Pellet Doses	Salt Doses	Parameters					
		PL (cm)	RL (cm)	SFW (g)	RFW (g)	SDW (g)	RDW (g)
Control	0	32.00	24.50 g	1.68 a	0.99 f	0.62 a	0.13 e
	25	29.50	27.00 f	1.30 de	1.20 e	0.47 d	0.18 d
	50	27.00	27.50 e	1.28 ef	1.26 de	0.44 e	0.19 d
	100	28.25	29.50 b	0.96 hi	1.62 a	0.44 e	0.23 a
Control mean		29.18 A	27.12 A	1.30	1.26 A	0.49 A	0.18 A
1%	0	28.00	24.00 g	1.51 ab	1.22 de	0.54 b	0.18 d
	25	27.00	27.00 f	1.38 b–d	1.27 de	0.49 c	0.19 d
	50	25.00	27.25 ef	1.34 c–e	1.34 c	0.49 c	0.20 c
	100	27.25	30.50 a	1.31 de	1.39 b	0.46 d	0.22 b
1% mean		26.81 B	27.18 A	1.38	1.30 A	0.49 A	0.19 A
2%	0	28.75	24.50 g	1.61 a	1.29 c–e	0.54 b	0.19 cd
	25	24.25	27.25 ef	1.44 b	1.30 cd	0.52 b	0.18 d
	50	23.50	28.00 d	1.45 b	1.30 cd	0.53 b	0.18 d
	100	23.75	28.50 c	0.93 i	1.33 c	0.41 f	0.22 b
2% mean		25.06 BC	27.06 A	1.35	1.30 A	0.49 A	0.19 A
4%	0	24.75	20.75 j	1.15 fg	0.82 h	0.30 g	0.09 g
	25	23.75	20.50 j	1.04 gh	0.86 gh	0.24 h	0.10 g
	50	21.25	21.50 i	1.41 bc	0.91 fg	0.30 g	0.11 f
	100	23.50	23.00 h	1.43 b	1.39 b	0.43 e	0.19 d
4% mean		23.31 C	21.43 B	1.25	0.99 B	0.31 B	0.12 B
Salt doses mean	0	28.37 a	23.43 c	1.48 a	1.07 c	0.50 a	0.14 c
	25	26.12 b	25.43 b	1.28 ab	1.15 bc	0.43 b	0.16 b
	50	25.68 b	26.06 b	1.36 a	1.20 b	0.44 b	0.16 b
	100	24.18 b	27.87 a	1.15 b	1.43 a	0.43 b	0.21 a
CV (%)		8.76	3.88	14.83	9.8	6.82	9.94
WP		**	**	ns	**	**	**
S		**	**	**	**	**	**
WP × S		ns	*	**	**	**	**

CV—coefficient of variation; WP—significance of wool pellet doses; S—significance of salt doses; WP × S—significance of wool pellet salt interaction; PL—plant length; RL—root length; SFW—stem fresh weight; SDW—steam dry weight; RFW—root fresh weight; RDW—root dry weight; * significant at *p* < 0.05; ** significant at *p* < 0.01; and ns—non-significant. The values within each row denoted by different lowercase letters indicate significant differences between salt dose treatments, while the values denoted by different capital letters indicate significant differences between Wool pellet dose treatments.

**Table 3:** The effect of wool pellets and salt doses on some physiological and biochemical characteristics in soybean.

Wool Pellet Doses	Salt Doses	Parameters					
		LT (°C)	LA (cm^2^)	NBI (dx)	Chlorophyll (dx)	Flavonoid (dx)	MDA (nmol/g FW)
Control	0	19.85	9.42 e	102.13 h	40.48 b–d	0.39	18.09 kl
	25	20.00	8.27 f	73.88 l	34.55 fg	0.46	19.43 j
	50	20.20	7.43 g	91.43 k	30.88 h	0.43	20.24 i
	100	20.30	6.82 i	59.25 n	28.93 i	0.50	24.85 c
Control mean		20.08 C	7.98 D	81.66 D	33.70 B	0.44 A	20.65 B
1%	0	20.40	12.23 b	117.28 e	50.43 a	0.36	18.69 jk
	25	20.35	10.34 c	96.05 i	41.30 bc	0.42	23.40 de
	50	20.60	9.96 c	74.23 l	35.58 f	0.41	24.61 c
	100	20.80	8.42 f	66.68 m	37.30 e	0.49	26.14 b
1% mean		20.53 B	10.23 A	88.55 C	41.15 A	0.41 A	23.21 A
2%	0	21.50	13.46 a	96.70 i	40.05 b-d	0.40	21.51 h
	25	21.45	8.97 e	122.70 d	39.50 c–e	0.43	22.51 g
	50	21.45	8.41 f	113.83 f	42.20 b	0.42	22.85 fg
	100	22.45	7.04 h	111.53 g	37.35 e	0.43	23.30 e
2% mean		21.71 A	9.46 B	111.18 B	39.77 A	0.42 A	22.54 A
4%	0	21.35	12.08 b	141.48 b	39.50 b–e	0.27	17.24 l
	25	21.40	10.38 c	125.68 c	40.23 b–d	0.33	23.95 d
	50	21.40	7.01 hi	144.43 a	33.75 g	0.36	23.11 ef
	100	21.35	4.93 j	94.45 j	38.55 de	0.42	26.51 a
4% mean		21.37 A	8.59 C	126.50 A	38.00 A	0.34 B	22.70 A
Salt doses mean	0	20.77	11.79 a	114.39 a	42.61 a	0.35 c	18.88 c
	25	20.80	9.48 b	104.57 b	38.89 b	0.40 b	22.32 b
	50	20.91	8.20 c	105.97 b	35.6 b	0.40 b	22.70 b
	100	21.22	6.79 d	82.97 c	35.53 b	0.45 a	25.19 a
CV (%)		2.12	7.61	2.09	10.19	9.92	4.78
WP		**	**	**	**	**	**
S		ns	**	**	**	**	**
WP × S		ns	**	**	*	ns	**

CV—coefficient of variation; WP—significance of wool pellet doses; S—significance of salt doses; WP × S—significance of wool pellet salt interaction; LT—leaf temperature; LA—leaf area; NBI—nitrogen balance index, MDA—malondialdehyde; FW—fresh weight; * significant at *p* < 0.05; ** significant at *p* < 0.01; and ns—non-significant. The values within each row denoted by different lowercase letters indicate significant differences between salt dose treatments, while the values denoted by different capital letters indicate significant differences between Wool pellet dose treatments.

**Table 4:** The effect of wool pellets and salt doses on some biochemical characteristics in soybean.

Wool Pellet Doses	Salt Doses	Parameters			
		TAA (µmol TE/g)	TPC (mg GAE/g)	Carotenoid (μg/g FW)	MDA (nmol/g FW)
Control	0	14.86	115.50 d	0.72 h	18.09 kl
	25	16.91	127.42 c	3.11 a	19.43 j
	50	17.03	130.35 b	2.33 d	20.24 i
	100	19.94	143.35 a	3.00 b	24.85 c
Control mean		17.18 A	129.16 A	2.29 A	20.65 B
1%	0	10.94	96.57 f	0.25 l	18.69 jk
	25	12.32	98.92 f	1.66 f	23.40 de
	50	13.75	109.92 e	2.71 c	24.61 c
	100	13.96	111.07 e	2.07 e	26.14 b
1% mean		12.74 B	104.12 B	1.67 B	23.21 A
2%	0	8.22	61.57 i	0.33 k	21.51 h
	25	9.24	62.42 i	0.80 g	22.51 g
	50	10.86	62.85 i	0.63 j	22.85 fg
	100	10.46	64.78 h	0.28 l	23.30 e
2% mean		9.69 C	62.91 C	0.43 C	22.54 A
4%	0	6.07	60.85 i	0.74 h	17.24 l
	25	7.44	61.21 i	0.17 m	23.95 d
	50	8.50	66.21 gh	0.13 n	23.11 ef
	100	8.89	66.92 g	0.67 i	26.51 a
4% mean		7.72 D	63.80 C	0.43 C	22.70 A
Salt doses mean	0	10.02 d	83.62 d	0.43 c	18.88 c
	25	11.47 c	87.49 c	1.44 b	22.32 b
	50	12.53 b	92.34 b	1.45 b	22.70 b
	100	13.31 a	96.53 a	1.51 a	25.19 a
CV (%)		7.73	4.65	1.68	4.78
WP		**	**	**	**
S		**	**	**	**
WP × S		ns	**	**	**

CV—coefficient of variation; WP—significance of wool pellet doses; S—significance of salt doses; WP × S—significance of wool pellet salt interaction; TAA—total antioxidant activity; TPC—total phenolic content; MDA—malondialdehyde; TE—trolox equivalent; GAE—gallic acid equivalent; FW—fresh weight ** significant at *p* < 0.01; and ns—non-significant. The values within each row denoted by different lowercase letters indicate significant differences between salt dose treatments, while the values denoted by different capital letters indicate significant differences between Wool pellet dose treatments.

## Data Availability

Data are contained within the article.
